# Voltammetric Determination of Cocaine in Confiscated Samples Using a Carbon Paste Electrode Modified with Different [UO_2_(X-MeOsalen)(H_2_O)] · H_2_O Complexes

**DOI:** 10.3390/s130607668

**Published:** 2013-06-14

**Authors:** Laura Siqueira de Oliveira, Ana Paula dos Santos Poles, Marco Antonio Balbino, Matheus Manoel Teles de Menezes, José Fernando de Andrade, Edward Ralph Dockal, Heloísa Maria Tristão, Marcelo Firmino de Oliveira

**Affiliations:** 1 Departamento de Química, Faculdade de Filosofia, Ciências e Letras de Ribeirão Preto, Universidade de São Paulo, Ribeirão Preto 14040-901, São Paulo, Brasil; E-Mails: lauraolliver@yahoo.com.br (L.S.O.); appoles@hotmail.com (A.P.S.P.); marco.balbino@rocketmail.com (M.A.B.); matheusmtmenezes@gmail.com (M.M.T.M.); jfdandra@ffclrp.usp.br (J.F.A.); 2 Centro de Ciências Exatas e de Tecnologia, Departamento de Química, Universidade Federal de São Carlos, São Carlos 13565-905, São Paulo, Brasil; E-Mail: dockal@dq.ufscar.br; 3 Núcleo de Perícias Criminalísticas de Ribeirão Preto, Superintendência de Polícia Técnico-Científica do Estado de São Paulo, Ribeirão Preto14015-040, São Paulo, Brasil; E-Mail: helotri@hotmail.com

**Keywords:** forensic chemistry, cocaine, Schiff base complexes, chemically modified electrodes, cyclic voltammetry

## Abstract

A fast and non-destructive voltammetric method to detect cocaine in confiscated samples based on carbon paste electrode modified with methoxy-substituted N,N'-ethylene-bis(salcylideneiminato)uranyl(VI)complexes, [UO_2_(X-MeOSalen)(H_2_O)].H_2_O, where X corresponds to the positions 3, 4 or 5 of the methoxy group on the aromatic ring, is described. The electrochemical behavior of the modified electrode and the electrochemical detection of cocaine were investigated using cyclic voltammetry. Using 0.1 mol·L^−1^ KCl as supporting-electrolyte, a concentration-dependent, well-defined peak current for cocaine at 0.62 V, with an amperometric sensitivity of 6.25 × 10^4^ μA·mol·L^−1^ for cocaine concentrations ranging between 1.0 × 10^−7^ and 1.3 × 10^−6^ mol·L^−1^ was obtained. Chemical interference studies using lidocaine and procaine were performed. The position of the methoxy group affects the results, with the 3-methoxy derivative being the most sensitive.

## 1. Introduction

**Figure 1.**
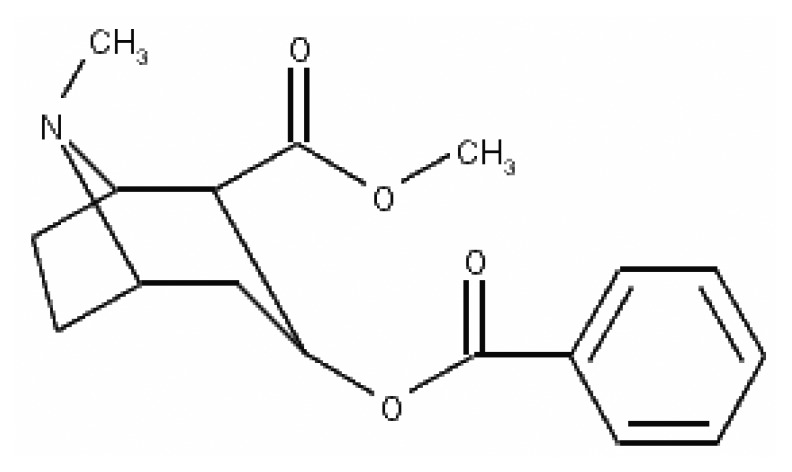
Chemical structure of cocaine.

Cocaine (Figure 1) is the main alkaloid extracted from *Erythroxylum coca*. In the early twentieth century, it was used as a component of tonics and beverages. Today, however, it is almost exclusively associated with its misuse, which poses great health risks and can even lead to death. [1]. Cocaine acts as a local anesthetic and stimulates the central nervous system, leading to increased alertness and euphoria [2]. These effects stem from the ability of cocaine to block synaptic dopamine reuptake. However, this alkaloid also blocks norepinephrine and serotonin reuptake, so chronic cocaine use modifies these neurotransmitter systems [3].

Cocaine increases heart rate and blood pressure, culminating in heightened arousal, improved performance in tasks requiring attention and caution, and feelings of confidence and well-being [3]. Cocaine abuse damages the cardiovascular and neurological systems, as well as the liver [4]. High doses produce euphoria and chronic use leads psychological disorders such as paranoia, irritability, and violent behavior [3].

In forensic analysis, cocaine is detected by chromatographic techniques such as GC–MS, HPLC, and LC–MS, for which the literature reports good accuracy, scientific robustness, and low sensitivity [5]. Electrochemical techniques constitute an important analytical tool in chemical analyses and are often used in the pharmaceutical, industrial, and clinical fields, as well as forensic studies [6–8]. Oye *et al.* developed a new detection method based on the electrochemical behavior of cocaine in a non-aqueous medium. These authors employed a platinum disk electrode chemically modified with a cobalt hexacyanoferrate film to determine cocaine in confiscated samples [9]. Thus, this work presents better results because there is a significant increase in amperometric sensitivity.

Electrochemical methods have also been used to analyze other illicit drugs, such as hemp. Balbino *et al.* investigated the electrochemical behavior of delta^9^-tetrahydrocannabinol (Δ^9^-THC), the psychoactive substance in hemp, by cyclic and linear sweep voltammetry using a glassy carbon working electrode [10]. Electrochemical techniques offer several advantages: (i) they are sensitive, accurate, and precise; (ii) they enable one to work with a large range, relative instrumentation and materials; (iii) they can be applied to colored materials and samples containing dispersed solid particles [7,8]; (iv) they provide rapid response; (v) and they allow for analysis of the irreversible electrooxidation of the tertiary amine present in the cocaine molecule [11].

This process involves abstraction of a lone electron pair from the amine nitrogen, followed by rapid proton loss, to form a neutral radical that loses an electron and undergoes hydrolysis to a secondary amine and a ketone [12].

The choice of electrode modifier for a chemically modified electrode is crucial. Gold, platinum, glassy carbon and carbon paste film are some of the conventional electrode materials [13]. Chemically modified carbon paste electrodes have received considerable attention and have been increasingly used to measure a variety of organic compounds of biological and pharmaceutical interest [14]. These modified electrodes are inexpensive, easy to manufacture, display easily renewable surfaces, and low background current, are compatible with various types of modifiers, and allow for work in a wide potential range [14–20].

Schiff base chemistry has attracted a lot of interest because these are important intermediates in the synthesis of certain bioactive compounds such as β-lactams [21]. These compounds exhibit significant biological activity, such as antifungal [22], antimicrobial [23,24], antibacterial [22] and antitumor actions [25]. Schiff bases have a number of applications: they can be used as electrode surface modifiers [26–41], as selective chelating titrants for copper (II) [32], as extraction reagents for spectrophotometric determination of copper (II) [33], as chromogenic reagents for the determination of nickel in food samples [34] and cobalt (II) [35], as reagents for extraction of ion pairs of divalent cations [36], and as complexing agents for “online” extraction/preconcentration of copper and lead in atomic absorption spectrometry-flow injection [37]. Schiff bases can also be employed in films [38,39] that protect copper against corrosion [40,41]. However, the use of Schiff base complexes as electrode modifiers for the detection of drugs of abuse, particularly cocaine, has not yet been reported.

**Figure 2.**
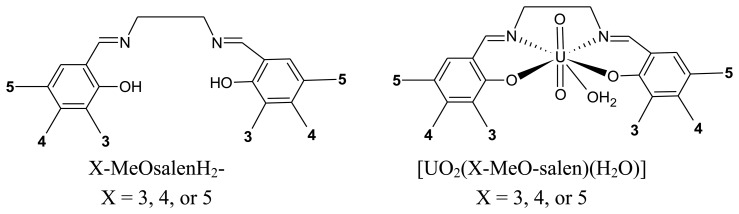
Structural representations of the ligands and complexes used in this study.

In the present work, the use of [UO_2_(L)(H_2_O)]·H_2_O complexes as surface modifiers for carbon paste electrodes for the electrochemical detection of cocaine is investigated. The uranyl(VI) complexes, [UO_2_(L)(H_2_O)]·H_2_O, of the tetradentate Schiff bases, *N*,*N'*-*bis*(3-methoxysalicylidene)-ethylenediamine, H_2_L^1^; *N*,*N'*-*bis*(4-methoxysalicylidene)-ethylenediamine, H_2_L^2^; and *N*,*N'*-*bis*(5-methoxysalicylidene) ethylenediamine, H_2_L^3^, (Figure 2), are prepared to investigated the effect of the position of the methoxy substituent on the sensitivity of the electrochemical detection of cocaine.

At this time, the interaction between the Schiff base complexes and cocaine has not been reported in the literature. There is evidence that the uranyl oxygen present in the Schiff base complex interacts with the carboxyl group of the cocaine molecule.

Considering the Schiff base complexes can be used as electrode modifiers for the analysis of pharmaceutical substances, there are no reports on their application for forensic purposes. Thus, this research aimed to develop carbon paste electrodes chemically modified with Schiff base complexes to determine cocaine in samples seized by the police using voltammetric techniques.

## 2. Experimental

### 2.1. Synthesis of Compounds

Elemental microanalyses, C, H, N, were performed by the Microanalytical Laboratory of the Departamento de Química of the Universidade Federal de São Carlos, São Carlos, São Paulo (Brazil) with a Faison EA 1108 CHNS-O instrument. Melting points were determined using a Marconi NA 301, Piracicaba-SP, Brazil. IR spectroscopy on KBr pressed discs (1% by weight) with a Bomem Michelson 102 FT-IR instrument (Zurich, Switzerland).

### 2.2. Preparation of the Schiff Bases

The Schiff bases were prepared in a similar manner [42,43] using the following procedure: to a stirred solution of 10 mmol of the appropriate substituted salicylaldehyde (Sigma-Aldrich, St. Louis, MO, USA) in ethanol was added dropwise an ethanolic solution of 5 mmol ethylenediamine (Sigma-Aldrich). This mixture was then heated for 2 h. Afterwards the mixture was cooled to room temperature, then for 24 h at 5 °C. The solid was filtered and washed with cold ethanol (5 mL at 5 °C) and dried over silica. The ligands were used without further purification.

#### 2.2.1. Ligand H2L^1^, N,N'-ethylene-bis(3-methoxysalcylideneimine), 3-MeOsalenH_2_

Yellow powder. Yield: 1.400 g, 85.3%. m. p.: 165–167 °C (uncorrected). Anal.. Calc. for C_18_H_20_N_2_O_4_: C, 65.8; H, 6.1; N, 8.5. Found: C, 65.4; H, 6.1; N, 8.3%. Selected IR bands (KBr cm^−1^): 1,633 s, ν_C=N_; 1,410 m, ν_C–N_; 1,295 m, ν_C–O_; 1,251 m, ν_C–O–C_.

#### 2.2.2. Ligand H_2_L^2^, N,N'-ethylene-bis(4-methoxysalcylideneimine), 4-MeOsalenH_2_

Yellow powder. Yield: 0.280 g, 42.7%. m. p.: 171–172 °C (uncorrected). Anal.. Calc. for C_18_H_20_N_2_O_4_: C, 65.8; H, 6.1; N, 8.5. Found: C, 65.6; H, 6.0; N, 8.4%. Selected IR bands (KBr·cm^−1^): 1,619 s, ν_C=N_; 1,396 m, ν_C–N_; 1,286 m, ν_C–O_; 1,223 m, ν_C–O–C_.

#### 2.2.3. Ligand H_2_L, N,N'-ethylene-bis(5-methoxysalcylideneimine), 5-MeOsalenH_2_

Yellow powder. Yield: 0.280 g, 42.7%. m. p.: 154–155 °C (uncorrected). Anal.. Calc. for C_18_H_20_N_2_O_4_: C, 65.8; H, 6.1; N, 8.5. Found: C, 65.7; H, 6.2; N, 8.4%. Selected IR bands (KBr·cm^−1^): 1,638 s, ν_C=N_; 1,587 m, ν_C=C_; 1,339 m, ν_C–N_; 1,275 m, ν_C–O_; 1,228 m, ν_C–O–C_.

### 2.3. Preparation of [UO_2_(L)(H_2_O)].H_2_O

To a boiling ethanol solution containing the appropriate ligand was added a solution of uranyl acetate dihydrate, UO_2_(C_2_H_3_O_2_)_2_·2H_2_O (0.706 g, 1.7 mmol, Merck, Darmstadt, Germany) in distilled water (40 mL) and four drops of glacial acetic acid. The orange-red solution was refluxed with stirring for 2 h. The resulting precipitate was collected by filtration, washed twice with distilled water (20 mL) and twice with ethanol (15 mL) and dried in a desiccator over silica at room temperature.

#### 2.3.1. [UO_2_(L^1^)(H_2_O)]·H_2_O, [UO_2_(3-MeOsalen)(H_2_O)] ·H_2_O

Red powder. Yield: 0.632 g, 58.8%. decomposition temperature 346–347 °C (uncorrected). Anal Calc. for C_18_H_22_N_2_O_8_U: C, 34.1; H, 5.1; N, 4.4. Found: C, 34.0; H, 5.2; N, 4.5%. Selected IR bands (KBr·cm^−1^): 1,626 s, ν_C=N_; 1,553 m, ν_C=C_; 1,399 m, ν_C–N_; 1293 m, ν_C–O_; 1,242 s, ν_C–O–C_; 900 s, ν_U=O_.

#### 2.3.2. [UO_2_(L^2^)(H_2_O)]·H_2_O, [UO_2_(4-MeOsalen)(H_2_O)]·H_2_O

Orange powder. Yield: 0.385 g, 35.8%. decomposition temperature 262–263 °C (uncorrected). Anal Calc. for C_18_H_22_N_2_O_8_U: C, 34.1; H, 5.1; N, 4.4. Found: C, 34.1; H, 5.0; N, 4.3%. Selected IR bands (KBr·cm^−1^): 1,613 s, ν_C=N_; 1,540 m, ν_C=C_; 1,389 m, ν_C–N_; 1,304 m, ν_C–O_; 1,216 s, ν_C–O–C_; 896 s, ν_U=O_.

#### 2.3.3. [UO_2_(L^3^)(H_2_O)]·H_2_O, [UO_2_(5-MeOsalen)(H_2_O)]·H_2_O

Orange-red powder. Yield: 0.486 g, 45.2%. decomposition temperature >365 °C (uncorrected). Anal Calc. for C_18_H_22_N_2_O_8_U: C, 34.1; H, 5.1; N, 4.4. Found: C, 34.0; H, 5.2; N, 4.5%. Selected IR bands (KBr·cm^−1^): 1631 s, ν_C=N_; 1,553 m, ν_C=C_; 1,385 m, ν_C–N_; 1,281 m, ν_C–O_; 1,231 s, ν_C–O–C_; 902 s, ν_U=O_.

### 2.4. Cocaine Purification

All the cocaine samples and standard solutions were obtained via a partnership with the criminal experts of the laboratory of toxicological analysis—Institute of Criminalistics, Ribeirão Preto, São Paulo, Brazil. Solutions of cocaine from confiscated samples (seized cocaine samples) were prepared according with literature [9]. Aliquots of the water-soluble cocaine hydrochloride samples were pretreated with sodium bicarbonate solution, to remove HCl. [9]. Hydrochloride-free cocaine is insoluble in water, so this product was removed by filtration, rinsed with deionized water, dried, and dissolved in KCl 0.1 mol·L^−1^ supporting-electrolyte solution.

### 2.5. Preparation of the Supporting-Electrolyte and Analyte (Cocaine)

An aliquot of 1.8 g of KCl (PA Acros Organics, Geel, Belgium) was added to a 250 mL flask containing distilled water, to give the supporting electrolyte KCl 0.1 mol·L^−1^. Procaine (Sigma-Aldrich) and lidocaine (Sigma-Aldrich) solutions (10 mL, 1 × 10^−2^ mol·L^−1^) were prepared and acidified until pH 2 with of HCl (PA, Merck), to ensure the solubility.

### 2.6. Preparation of Chemically Modified Electrodes

The chemically modified electrodes were prepared using three different Schiff base complexes: [UO_2_(3-MeOsalen)(H_2_O)] ·H_2_O, [UO_2_(4-MeOsalen)(H_2_O)] ·H_2_O, and [UO_2_(5-MeO-salen)(H_2_O)] ·H_2_O. The electrodes contained the following graphite mass/complex mass ratio: 75:25, 80:20, 85:15, 90:10, 95:5. First, 50 μL of nujol mineral oil was added to each composition, for agglutination. The mixture was homogenized under stirring with hexane and the solvent was removed in a rotary evaporator. Transducers were prepared using hollow cylindrical glass tubes with internal diameter of 1 mm containing a gold electric contact between the paste and the copper connection. The space filled by the copper wire acted as the external electrical contact. The paste was filled with about 1 cm of each investigated mixture. After the working electrode was ready, it was set up the in electrochemical cell. 0.1 mol·L^−1^ KCl supporting electrolyte (4 mL) was added to the cell and a nitrogen gas flow was applied for 15 minutes, to remove electroactive oxygen. The electrochemical cell consisted of carbon paste working electrode, an auxiliary platinum wire electrode, and the reference Ag/AgCl electrode.

### 2.7. Voltammetric Measurements

Cyclic voltammetry was conducted on an AutoLabIII potentiostat (Metrohm, Utrecht, Netherlands) coupled to a microcomputer. The potential scans were performed between −0.4 V and 1.2 V, at a speed of 100 mV·S^−1^. Voltammograms at different concentrations of cocaine were recorded by the standard addition method. The same procedure was followed for procaine and lidocaine analysis during the study of interferents.

## 3. Results and Discussion

### 3.1. Stability Studies of the Schiff Base Complex Films

Carbon paste electrodes were chemically modified with the Schiff base complexes [UO_2_(3-MeOSalen)(H_2_O)] ·H_2_O, [UO_2_(4-MeOSalen)(H_2_O)] ·H_2_O, and [UO_2_(5-MeOSalen)(H_2_O)] ·H_2_O. The films have showed good stability during the first 20 cycles at 100 mV·s^−1^. A decrease of current was not observed in these measurements.

### 3.2. Electrochemical Behavior of [UO_2_MeoSalen(H_2_O)]·H_2_O Carbon Paste

The uranyl(VI) complexes undergo reduction on electrode surface [44]. A redox reaction corresponds to the oxidation of the metal species chelated with the Schiff base, as follows:

$${{\text{UO}}_{2}}^{2+}\Leftrightarrow {{\text{UO}}_{2}}^{4+}+{\text{H}}^{+}+2{\text{e}}^{-}$$

### 3.3. Influence of the Composition of the Carbon Paste Containing [UO_2_(3-MeOsalen)(H_2_O)].H_2_O

Modifiers should have good sensitivity for the analyte. However, their proportion in the electrode may increase or decrease their affinity for the analyte. In this work we carried out voltammetry in KCl 0.1 mol·L^-1^ aqueous solution. As supporting-electrolyte, using as electrode modified with [UO_2_(3-MeOsalen)(H_2_O)] ·H_2_O, 75% graphite; 25% modifier, which was the ratio that gave the best electroanalytical response.

### 3.4. Influence of Cocaine Concentration

The working carbon paste electrode modified with [UO_2_(3-MeOsalen)(H_2_O)] ·H_2_O indicated a significantly increase of the amperometric sensitivity. Thus, we were able determine cocaine in μmol·L^−1^ using seized samples of cocaine. We obtained linear sweep (LSV) and cyclic (CV) by successively adding aliquots to the electrochemical cell, which analytical curve furnished the linearity over the studied concentration range from 1 × 10^−7^ to 1.3 × 10^−6^ mol·L^−1^ was good. The correlation coefficient (r) was 0.97 with a standard deviation (SD) of 0.005 μA. The equation for cocaine determination was:

$$\text{ipa}=0.24\;\mu \text{A}+4.6\times 10^{4}\mu \text{A}/\text{mol}\cdot {\text{L}}^{–1}\;[\text{cocaine}]$$

Using the relation 3 SD/m and 10 SD/m (where m is the amperometric sensitivity of the curve), we obtained a limit of detection (LOD) of 0.326 μmol·L^−1^ and a limit of quantification (LOQ) of respectively, using cocaine concentration ratio remained as 1.1 × 10^−6^ mol·L^−1^. Therefore, the carbon paste electrode modified with [UO_2_(3-MeOsalen)(H_2_O)]·H_2_O has high analytical sensitivity for cocaine. We determined the parameters Epa, Epc, ipa and ipc for the peaks observed between 0.5 and 0.6 V (Table 1). The values confirm that increases linearly. Epa rose by 0.63 V for scan rates between 35 and 100 mV·s^−1^. A scan rate of 100 mV·s^−1^ gave better ipa. The Epa/Epc and ip/v^1/2^ ratios indicate the reaction mechanism. The lower the Epa/Epc ratio values suggested that a reversible process occurred, because Ep (Epa-Epc) was 56 mV.

**Figure 3.**
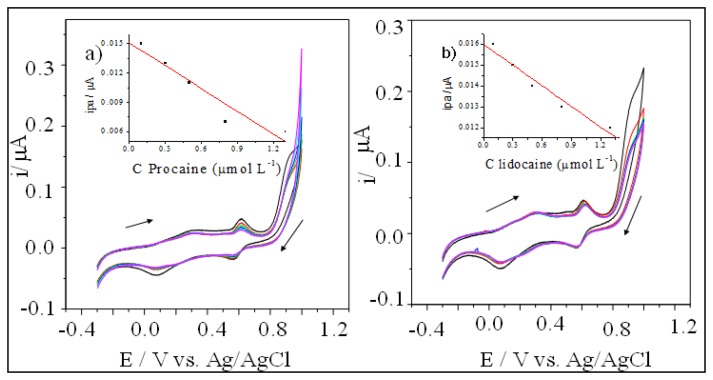
(**a**) Influence of the concentration of procaine and (**b**) lidocaine standard samples on the electrochemical response of the electrode modified with [UO_2_(3-MeO-salen)(H_2_O)]·H_2_O.

Procaine and lidocaine are widely used to adulterate cocaine samples. The presence of these interferents decreased the anodic peaks registered using the electrode modified with [UO_2_(3-MeOsalen) (H_2_O)]·H_2_O (Figure 3).

**Figure 4.**
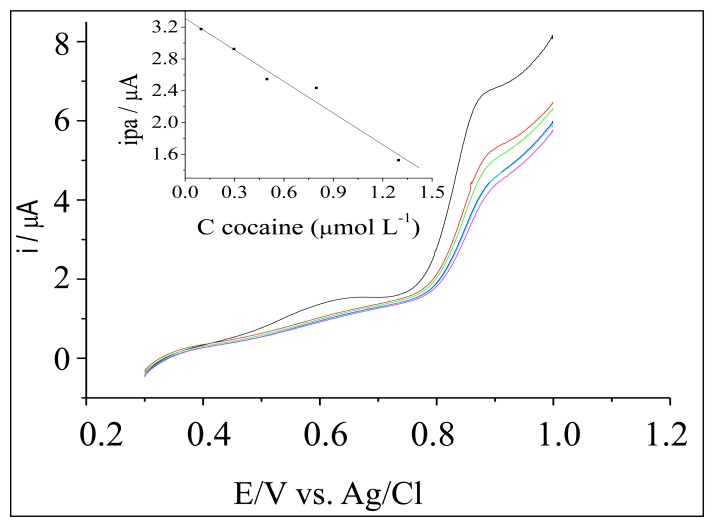
Influence of the concentration cocaine standard samples in the electrochemical response of the electrode modified with [UO_2_(5-MeO-salen)(H_2_O)]·H_2_O.

**Figure 5.**
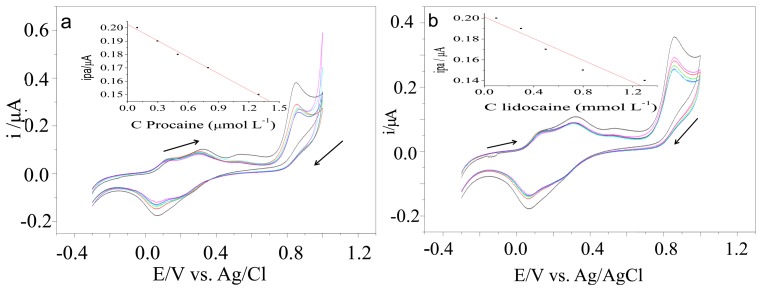
Influence of the concentration of procaine (**a**) and lidocaine (**b**) standard samples on the electrochemical response of the electrode modified with [UO_2_(5-MeO-salen)(H_2_O)]·H_2_O.

We used the same instrumental parameters to study the electrode modifiers such [UO_2_(4-MeOsalen)(H_2_O)]·H_2_O and [UO_2_(5-MeO-salen)(H_2_O)]·H_2_O. The carbon paste working electrode modified with [UO_2_(4-MeOsalen)(H_2_O)]·H_2_O in different ratios (75–95% graphite and 5–25% of the modifier) was not active for cocaine, lidocaine, or procaine. The LSV recorded with the carbon paste electrode modified with [UO_2_(5-MeO-salen)(H_2_O)]·H_2_O. at a 95%:5% graphite/modifier (Figure 4) ratio revealed reduced peak current for oxidation upon successive addition of the standard cocaine solution (Table 2). For procaine, and lidocaine analyses, we employed a CV technique (Figure 5).

The electrodes modified the with the [UO_2_(3-MeOsalen)(H_2_O)] ·H_2_O have adequate specificity for cocaine analysis. Electrodes modified with [UO_2_(4-MeOsalen)(H_2_O)] ·H_2_O and [UO_2_(5-MeO-salen)(H_2_O)] ·H_2_O do not display a satisfactory electrochemical activity profile to distinguish between the different studied analytes.

We tested the proposed transducer in the presence of cocaine, lidocaine, and procaine. Using cyclic voltammetry, results indicated that lidocaine and procaine samples showed lower current peaks than cocaine in the same potential range. Therefore, the proposed system provides unequivocal cocaine identification in seized samples containing these interferents.

## 4. Conclusions

Voltammetric analysis of cocaine using carbon paste electrode modified with the Schiff base complex, [UO_2_(3-MeOsalen)(H_2_O)] ·H_2_O indicated the specific electrochemical activity of this electrode, without interference of lidocaine or procaine. The peak current at 0.62 V varies linearly with the cocaine concentration. This device has an amperometric sensitivity of 4.6 × 10^4^ μA/mol·L^−1^ in the working range of 1.0 × 10 ^−7^ to 1.3 × 10^−6^ mol·L^−1^ cocaine, indicating that the transducer can be applied for quantitative analysis of cocaine. The results point to the importance of the position of the ethoxy group in the molecule of the modifier. To summarize, Schiff base complexes can be used to develop chemically modified electrodes for the detection of organic substances of forensic interest. The electrode modified with [UO_2_(3-MeOsalen)(H_2_O)] ·H_2_O is potentially useful in the forensic field and can be employed in a more specific methodology for the preliminary testing of cocaine in drug samples.

## Figures and Tables

**Table 1.** Voltammetric parameters obtained at different custom potential scan rates for the carbon paste electrode modified with [UO_2_(3-MeO-salen)(H_2_O)] ·H_2_O.

**V (mV/s)**	**V^1/2^**	**Epa (mV)**	**Epc (mV)**	**Ipa (μA)**	**Ipc (μA)**
5	2.24	635	594	0.08	0.16
10	3.16	630	588	0.14	0.24
25	5.00	632	581	0.29	0.46
35	5.92	636	580	0.41	0.61
50	7.07	637	575	0.53	0.75
75	8.66	636	568	0.63	0.99
100	10.00	637	566	0.76	1.38

**Table 2.** Voltammetric parameters obtained with successive addition of standard cocaine solution to the carbon paste electrode modified with [UO_2_(5-MeO-salen)(H_2_O)]·H_2_O.

**Addition of Cocaine (μL)**	**Concentration of Cocaine (mol·L^−1^)**	**Ipa (μA)**
No addition	0	4.50
10	1 × 10^−7^	3.17
30	3 × 10^−7^	2.92
50	5 × 10^−7^	2.54
80	8 × 10^−7^	2.43
130	1.3 × 10^−6^	1.52
